# A dataset on survey designs and quality of social and behavioral science surveys during the COVID-19 pandemic

**DOI:** 10.1038/s41597-024-03475-x

**Published:** 2024-06-12

**Authors:** Tobias Gummer, Thomas Skora, Karolina von Glasenapp, Elias Naumann

**Affiliations:** 1 GESIS – Leibniz Institute for the Social Sciences, Mannheim, Germany; 2 University of Mannheim, School of Social Sciences, Mannheim, Germany

**Keywords:** Interdisciplinary studies, Sociology

## Abstract

In the social and behavioral sciences, surveys are frequently used to collect data. During the COVID-19 pandemic, surveys provided political actors and public health professionals with timely insights on the attitudes and behaviors of the general population. These insights were key in guiding actions to fight the pandemic. However, the data quality of these surveys remains unclear because systematic knowledge about how the survey data were collected during the COVID-19 pandemic is lacking. This is unfortunate, since decades of survey research have shown that survey design impacts data. Our Survey Data Collection and the COVID-19 Pandemic (SDCCP) project deals with this research gap. We collected rich metadata on survey design for 717 social and behavioral science surveys carried out in Germany during the first two years of the COVID-19 pandemic. In this data descriptor, we present a unique resource for a systematic assessment of the survey data collection practices and quality of surveys conducted in Germany during the COVID-19 pandemic.

## Background & Summary

During the COVID-19 pandemic, social and behavioral data provided political actors and public health professionals with an empirical basis for guiding their actions in fighting the pandemic^1^. In the social and behavioral sciences, surveys are an important technique to gather data on the attitudes and behavior of the (general) population. Consequently, examples of the survey data collected during the COVID-19 pandemic include specialized fit-for-purpose surveys such as the COVIDiSTRESS Global Survey^2^ and COVID-19-adjusted modules/and supplemental waves in existing and on-going general-purpose surveys such as the Mannheim Corona Study (MCS) in the German Internet Panel (GIP)^3^, the SOEP-CoV as part of the German Socio-Economic Panel (SOEP)^4^, and the tracking of COVID-19 effects in the Understanding America Study (UAS)^5^.

Survey data collection did not remain unaffected by the sudden COVID-19 outbreak in early 2020 as contributions in a special issue of Survey Research Methods detail^6^. In response to the pandemic, non-pharmaceutical interventions were put in place such as contact restrictions, stay-at-home orders, and closed public services/stores^7^. In response to these interventions, surveys began to adapt their designs to ensure that data could be collected. For example, to conclude their fieldwork that was interrupted by the COVID-19 outbreak, the German Family Panel (pairfam) switched from a face-to-face mode (F2F) to computer assisted telephone interviewing (CATI)^8^. Similarly, the Panel Study of Income Dynamics (PSID) also switched from F2F to CATI for their Transition into Adulthood Supplement (TAS-19)^9^. The Family Research and Demographic Analysis (FReDA) panel switched their recruitment mode from F2F to self-administered modes (mail, web)^8^. In line with these shifts away from F2F, the Understanding Society panel also abandoned the F2F mode^10^.

Despite the challenges imposed by the COVID-19 pandemic, survey research was able to continue data collection. In Germany, the German Data Forum (RatSWD) lists and briefly describes around 300 COVID-19-related surveys^11^. As detailed above, a few large-scale surveys described the necessary changes made to their survey design and operations protocols to ensure that fieldwork could be conducted. However, in general it remains an open question as to how survey data were collected during the COVID-19 pandemic, especially when considering the plethora of surveys that were not large-scale operations that had the resources to report their struggles and design decisions. In other words, it remains unclear which survey designs were used during this period to collect data.

Decades of survey research have highlighted that survey design impacts the quality of collected data^12,13^. Moreover, the COVID-19 pandemic itself may have affected survey measures in the form of a period effect. First, nonresponse bias may have been introduced into the data due to an expected correlation between COVID-19 infections and survey participation^14,15^. Second, the use of retrospective questions to assess respondent characteristics before the outbreak may have introduced measurement error at the respondent level^16^. Unfortunately, researchers not only lack knowledge about how surveys were collected during the COVID-19 pandemic but also, as a consequence, the data quality of this important basis for research, policy consultation, and political actions remains unaccounted for.

The Survey Data Collection and the COVID-19 Pandemic (SDCCP) project was funded by the German Federal Ministry for Education and Research (BMBF) from 2021 to 2025 to deal with the existing research gap on survey data collection practices and data quality. For this purpose, we investigate how survey data were collected during the pandemic and analyze the quality of the data that was used to consult political actors and conduct research.

To answer these research questions, as part of the SDCCP project, we aimed to collect rich metadata and information about survey design for all the social science surveys that were conducted in Germany between the COVID-19 outbreak in Germany from March 2020 and December 2021. We focused only on those surveys that targeted the German general population. As part of the project, we published the SDCCP dataset for re-use by research. This unique dataset is a systematic assessment of survey data collection practices in Germany during the COVID-19 outbreak (N = 717). It enables social science methodologists to assess questions of survey design and quality. In addition, the SDCCP dataset is a unique source of information on how scientific data were collected in a challenging environment. Moreover, this dataset can be used to supplement substantive analyses with survey design information. Enriching datasets with methodological information could be especially useful when aggregating data from different surveys that differ in how they collected data. Consequently, our goal with the present data descriptor is to detail our data collection approach and illustrate our dataset so to facilitate its use by other researchers.

## Methods

### Case selection

The SDCCP dataset includes information on the design of surveys conducted during the COVID-19 pandemic. To define which surveys to select for inclusion in our dataset, we established a list of survey eligibility criteria:

1. *Survey country:* Surveys eligible for inclusion in the dataset must have been conducted in Germany. Cross-national surveys also were considered eligible provided that a sub-sample was collected in Germany. In these cases, coding was restricted to the German sub-sample. Furthermore, eligible surveys had to target the entire country. We excluded surveys targeting only specific geographic areas (e.g., only certain federal states).
2. *Time period:* We only considered surveys with fieldwork starting between March 1, 2020 and December 31, 2021.
3. *Data type:* Eligible cases included academic quantitative surveys with voluntary participation.
4. *Topic:* Eligible surveys had to be conducted in the field of social sciences and/or health research.
5. *Target population:* Eligible surveys had to target individuals from the German general population. Surveys targeting very large population subgroups (e.g., individuals of a certain age range, students, or workers) also were considered eligible.

**Fig. 1:**
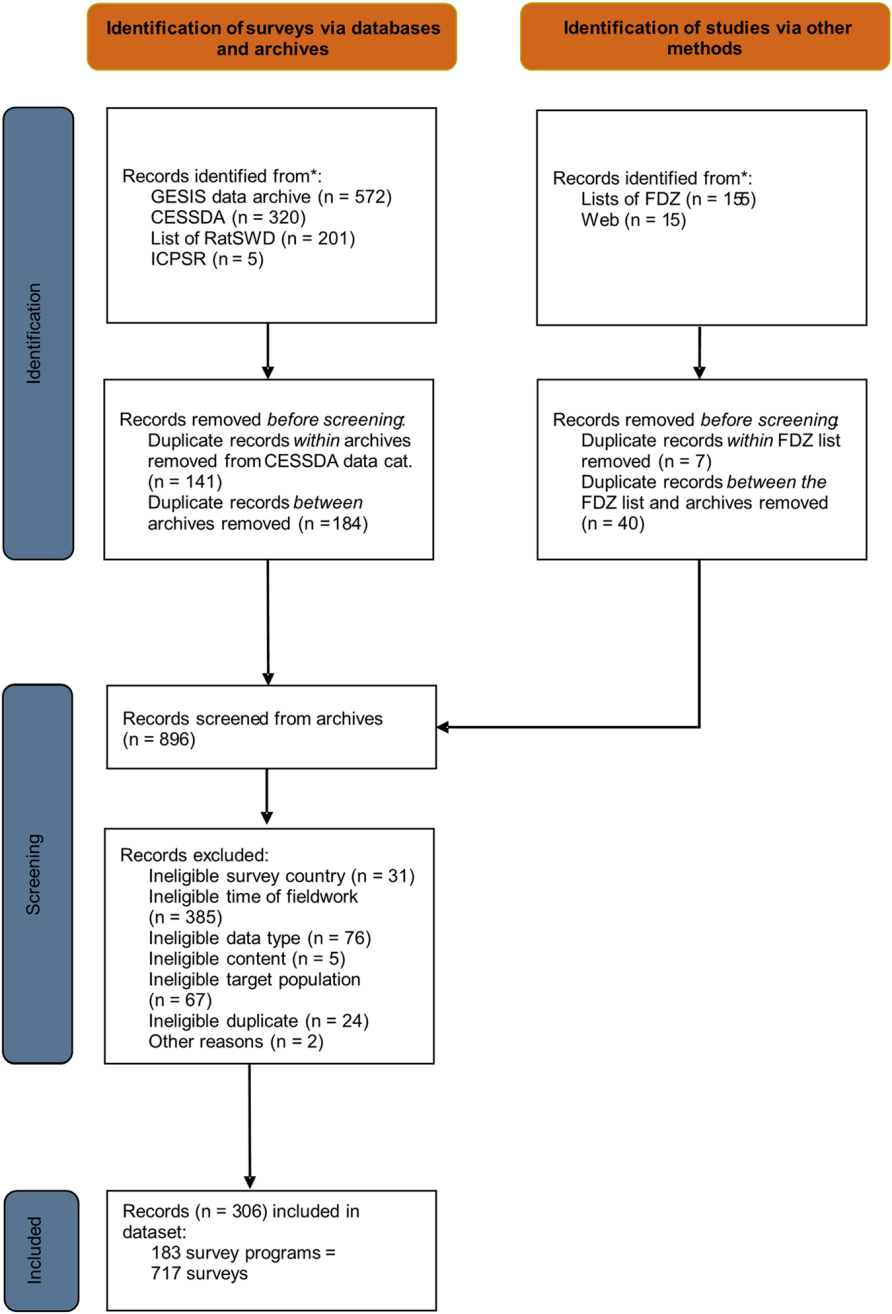
Survey selection (PRISMA) flow chart.

Based on these eligibility criteria, we conducted a three-step case selection process that we depicted as a flow diagram based on the PRISMA guidelines in Fig. 1^17^.

First, we identified potentially eligible surveys by searching the following repositories and lists of data collection efforts for surveys conducted in Germany during the relevant time period: GESIS Data Archive for the Social Sciences, Consortium of European Social Science Data Archives (CESSDA), RatSWD list of COVID-19 surveys, Inter-university Consortium for Political and Social Research (ICPSR), and data centres of the German Data Forum (FDZ). Our selection of repositories included the major German archive for quantitative social science data to which we added its European (CESSDA) and international counterparts (ICSPR) for completeness. To further incorporate surveys not covered by these repositories, we decided to include the above-mentioned lists to further enhance the completeness of our selection. Moreover, we searched for surveys that were mentioned in documentations, websites, or reports of the surveys we found in the repositories and lists but that were not included in the above-listed sources (category “web”). Our approach aimed to include as many surveys as possible in a systematic way, while also focusing on sources from which we were expecting to at least get some very basic and systematic information that was required to determine the eligibility of the cases. Given the extent of their content, we are convinced that the repositories and the RatSWD list cover the majority of social science surveys conducted during the COVID-19 pandemic in Germany. When available, we used filters in the repositories and lists that matched our eligibility criteria to adjust the scope of our searches. After the removal of duplicates, this step resulted in a list of 896 records.

Second, we screened the identified records for eligibility based on the publicly available information for each dataset (website, data documentation, method reports, etc.). For this purpose, we used our list of eligibility criteria in a hierarchical order, and we coded the first unfulfilled criterion as the reason for exclusion. As an illustration, a survey conducted in February 2020 with school staff did not fulfill criterion 2 (time period) or criterion 5 (target population), but we assigned its ineligibility only to criterion 2 due to its higher position in the hierarchy. If we could not determine a survey’s eligibility due to missing information, we did not include it in the dataset. We conducted the identification and screening process between September and November 2022. After the removal of ineligible records, 306 records remained.

Third, the remaining 306 records were coded what resulted in the SDCCP dataset. The final SDCCP dataset included 183 survey programs. We defined a *survey program* as having a common survey initiator and theme, and it may consist of multiple surveys (e.g., waves). In the SDCCP dataset, a *survey* is the unit of analysis (i.e., one row in the dataset). However, in a few cases (3.6% of all rows in the final dataset) no wave-specific information was available, but rather aggregated information referring to more than one wave. In these cases, we coded the survey program as one row incorporating the aggregated information we were able to obtain. To enable data users to identify these cases, we created a variable (v6) indicating for each row in the dataset how many individual surveys are represented by that row (see Usage Notes). In the final dataset, coding the 183 survey programs resulted in a sample size of 717 surveys.

### Coding scheme

The SDCCP dataset was designed to answer the project’s research questions related to survey design and data quality, and for reuse by scholars interested in research designs. For a holistic assessment of survey data quality, we relied on three well-established frameworks: the Total Survey Error (TSE) framework^13,18,19^, the Total Survey Quality (TSQ) framework^20^, and the FAIR Data Principles framework^21^. These frameworks differ in their definitions of data quality. On the one hand, the TSE framework understands data quality as the accuracy of survey statistics. The focus on accuracy corresponds with the quality perception of data producers. On the other hand, the FAIR Data Principles framework emphasizes the quality aspects that are important for data users, such as the availability of information on the study design and the availability of survey data. The Total Survey Quality framework combines both perspectives, since it considers accuracy and user-centered quality dimensions.

By combining the interests of data producers and data users, the coding scheme applied in the SDCCP dataset enables a multi-dimensional assessment of survey data quality with respect to the following dimensions:

- *Accuracy:* Accuracy can be assessed through the impact of key survey design features (e.g., sampling procedure, mode) on the TSE. For this purpose, our coding scheme includes variables to capture detailed information about the survey design used. We included the codes on which decisions were taken by the data producers regarding various survey design characteristics frequently discussed in textbooks on survey design^13,22,23^ and survey data reporting guidelines such as the AAPOR standards^24^.
- *Interpretability:* Interpretability refers to whether information about the survey design is available. A code capturing the presence of this information is provided for most variables in the dataset. Interpretability can be assessed by comparing these codes with the social science survey reporting standards (e.g., AAPOR standards^24^), guidelines for high-quality documentation of survey data^25^, and previous approaches to quality assessments of data documentation^26^.
- *Accessibility:* Accessibility can be assessed based on whether and when the survey data and the first results were made available to users. For this purpose, our coding scheme includes variables and codes on whether and when data and results were published.

Our coding scheme includes 121 variables that had to be coded for each survey. These codes are listed in our codebook.

### Coding procedure

The coding of data was performed between November 2022 and the end of May 2023 by two expert coders with a background in social science data collection methods. During the coding stages, interrater reliability was tested with randomly selected subsets of surveys (see Technical Validation). After each reliability test, the coders held a workshop and discussed discrepancies and decided together about cases in doubt. During the workshops, they also refined the coding scheme to remedy any shortcomings.

## Data Records

The SDCCP data are publicly available as a dataset in Stata format (.dta) and in CSV format (.csv) at the GESIS Data Archive for the Social Sciences^27^. The data download includes the dataset and the supporting documentation (e.g., coding scheme). The dataset covers a total of 717 surveys conducted as part of 183 survey programs. For each survey, a total of 121 variables were coded.

The SDCCP data are arranged in tabular format. Each row represents one survey, whereas each column represents one variable of the dataset (e.g., id, field period). Survey programs that fielded multiple waves or surveys during our observation period are represented as multiple rows in the dataset. The variables included in our dataset can be differentiated into three categories—technical variables, basic information, and quality variables:

- *Technical variables*: Technical variables enable the identification of the coders and the coded surveys. Multiple variables related to the survey ID enable analysis at various levels (e.g., a survey program ID for the most aggregated level and a wave-specific survey ID for a more detailed distinction).
- *Basic information*: Variables in this group provide information on the survey title, topic, its initiator, conductor, and funding.
- *Quality variables*: Quality variables cover various survey design features used in the above-described assessment of survey data quality (e.g., mode, sampling, sample size, availability of information and data).

Table 1 provides an overview of the coded characteristics by category. Detailed information on the variables can be found in the codebook provided as part of the dataset documentation in the GESIS Data Archive for the Social Sciences.

**Table 1:** An overview of variables included in the SDCCP dataset.

Technical variables	Basic information	Quality variables
Coder ID	Survey title	Temporal research design
Survey ID (at different levels of detail)	Survey DOI	Target population
	Survey website	Sampling procedure
	Topic	Survey length
	Survey initiator	Interview mode
	Survey conductor	Use of incentives
	Funding	Use of reminders
	Existence of the survey before the COVID-19 pandemic	Sample size
	Multiple countries included in the survey	Participation rate
	Fieldwork dates	Existence of weights
		Availability of the questionnaire, the data and the first results (if applicable: also the date of the first publication)

**Fig. 2:**
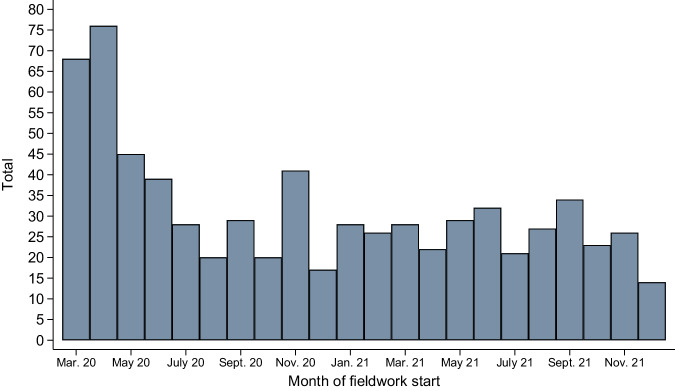
Number of fieldwork starts by calendar month.

Figure 2 shows the number of surveys by their month of fieldwork start. The comparatively high number in the first months illustrates the high demand for survey data, especially at the beginning of the COVID-19 pandemic.

## Technical Validation

At two points during the coding process, we conducted an assessment of *interrater reliability*, which is a numerical measure of the agreement between coders as to how to code the same data. High values indicate that the coding scheme is well specified and that most people would make the same coding decisions^28^. However, high agreement between coders also can result from chance alone. Therefore, various coefficients and indicators have been proposed that differ in their approaches to accounting for the chance in agreement^29–31^.

Based on the scale and empirical distribution of the variables in our coding scheme, we decided which coefficient and indicator to report. For assessing interrater agreement for nominally scaled items, we chose to apply Brennan and Prediger’s^32^ coefficient because these variables of our coding scheme were frequently found to have a highly uneven marginal distribution (i.e., ratings fall much more frequently under one category than the other). For several coefficients, including Cohen’s kappa and Krippendorff’s alpha, highly skewed distributions lead to reliability estimates that are counterintuitively low in the face of a high observed agreement, also known as the “prevalence problem”^30^ or “high agreement, but low reliability paradox”^29^. In contrast, Brennan and Prediger’s coefficient is not affected this way, since its concept of chance agreement is based on the assumption that if coders operated only by chance, a uniform distribution would arise, and, accordingly, merely considers the number of possible categories^31,33^. Brennan and Prediger’s coefficient is equivalent to the prevalence-adjusted and bias-adjusted kappa proposed by Byrt *et al*.^34^, also known as PABAK^31^, and Bennett *et al*.’s S coefficient^29,35^. To assess the interrater agreement with regard to the continuous scaled items, we calculated the intra-class correlation (ICC), a two-way model assessing absolute agreement^30^. Both coefficients were obtained using the Stata package KAPPAETC^31^.

For our interrater reliability analyses, a random 10% sample from all survey programs was double-coded (N = 20). For multi-wave survey programs, we only double-coded the first wave that was part of the dataset. We performed double coding in two rounds: once at the very beginning (N = 10) and once in the middle of the coding process (N = 10). This approach enabled us to discuss discrepancies and clarify the causes of conflicting interpretations among coders early in the coding process^28^.

We calculated all reliability coefficients once for the whole sample (N = 20), as well as separately for each assessment round. The latter approach enabled us to detect the impact of the clarification process after the first round on subsequent coding. As a summary indicator across all variables, we calculated the respective average of all single-item coefficients. Note that not all variables could be included in the analysis. In addition to string variables and technical variables, we excluded variables that were rarely coded due to filtering and had too few values (e.g., type and timing of applied incentives, timing of survey data releases). The variables that were included in the analyses, as well as the respective calculated coefficients, are listed in Table 2 (categorial variables) and Table 3 (continuous variables). Looking at Brennan and Prediger’s coefficient (Table 2), our analysis yielded an in-total average value of 0.87 (round 1: 0.85; round 2: 0.90). With respect to the ICC (Table 3), our analysis resulted in an in-total average value of 0.99 (round 1: 0.99; round 2: 0.94). All of these values indicate an excellent overall interrater reliability for both (considered) categorical variables and (considered) continuous variables^30,36,37^.

**Table 2:** Results of interrater reliability tests - categorial variables.

Var.	Label	Total			Round 1			Round 2		
		P_o_	k_q_	N_subj._	P_o_	k_q_	N_subj._	P_o_	k_q_	N_subj._
v18_1	Funding - DFG	1.00	1.00	20	1.00	1.00	10	1.00	1.00	10
v18_2	Funding - Ministry	0.80	0.70	20	0.80	0.70	10	0.80	0.70	10
v18_3	Funding - Private funding	1.00	1.00	20	1.00	1.00	10	1.00	1.00	10
v18_4	Funding organisation - Other	0.90	0.85	20	0.80	0.70	10	1.00	1.00	10
v20	Temporal research design	1.00	1.00	20	1.00	1.00	10	1.00	1.00	10
v21_1	LRD – Cross-section	0.80	0.70	15	0.71	0.57	7	0.88	0.81	8
v21_2	LRD – Panel	0.87	0.80	15	0.86	0.79	7	0.88	0.81	8
v23	Existence of the survey before the Covid-19 pandemic	1.00	1.00	20	1.00	1.00	10	1.00	1.00	10
v24	Multiple countries	0.95	0.93	20	1.00	1.00	10	0.90	0.85	10
v28	Probability/nonprob. sampling	0.95	0.93	20	0.90	0.85	10	1.00	1.00	10
v29	Use of an access panel	0.95	0.93	20	0.90	0.85	10	1.00	1.00	10
v32	Use of an existing survey as respondent pool	0.90	0.85	20	0.90	0.85	10	0.90	0.85	10
v39_1	SP - Probability simple random	0.90	0.85	20	0.80	0.70	10	1.00	1.00	10
v39_2	SP - Probability stratified	0.90	0.85	20	0.80	0.70	10	1.00	1.00	10
v39_3	SP - Probability cluster	0.90	0.85	20	0.80	0.70	10	1.00	1.00	10
v39_4	SP - Probability multistage	0.90	0.85	20	0.80	0.70	10	1.00	1.00	10
v39_5	SP - Nonprobability availability	0.80	0.70	20	0.80	0.70	10	0.80	0.70	10
v39_6	SP - Nonprobability purposive	0.95	0.93	20	0.90	0.85	10	1.00	1.00	10
v39_7	SP - Nonprobability respondent-assisted	0.90	0.85	20	0.90	0.85	10	0.90	0.85	10
v40	Use of quotas	0.83	0.75	12	0.71	0.57	7	1.00	1.00	5
v47_1	IM – CAPI	1.00	1.00	20	1.00	1.00	10	1.00	1.00	10
v47_2	IM – CATI	1.00	1.00	20	1.00	1.00	10	1.00	1.00	10
v47_3	IM – CAWI	1.00	1.00	20	1.00	1.00	10	1.00	1.00	10
v47_4	IM - Self-administered paper	1.00	1.00	20	1.00	1.00	10	1.00	1.00	10
v48	Use of incentives reported	0.90	0.85	20	1.00	1.00	10	0.80	0.70	10
v55	Use of reminders reported	0.94	0.91	17	1.00	1.00	9	0.88	0.81	8
v80	Existence of weights	0.70	0.55	20	0.70	0.55	10	0.70	0.55	10
v84	Questionnaire available	0.95	0.93	20	1.00	1.00	10	0.90	0.85	10
v86	Survey data available	0.85	0.77	20	1.00	1.00	10	0.70	0.55	10
v87	Existence of survey DOI	0.95	0.93	20	0.90	0.85	10	1.00	1.00	10
	**Average**	**0.92**	**0.87**		**0.90**	**0.85**		**0.93**	**0.90**	

*Notes:* P_o_ = percent agreement; k_q_ = Brennan and Prediger’s coefficient; N_subj_ = Number of compared subjects (i.e., surveys), LRD = Longitudinal research design, SP = Sampling procedure, IM = Interview mode. All variables exhibit three possible categories (including −9, i.e., “No information“).

**Table 3:** Results of interrater reliability tests - continuous variables.

Var.	Label	Total			Round 1			Round 2		
		P_o_	k_q_	N_subj._	P_o_	k_q_	N_subj._	P_o_	k_q_	N_subj._
v26	Target population – Min. age	1.00	1.00	11	1.00	1.00	5	1.00	1.00	6
v61 - v63	fieldwork start (dmy)	0.88	1.00	16	0.88	1.00	8	0.88	1.00	8
v64 - v66	fieldwork end (dmy)	0.93	1.00	15	0.88	1.00	8	1.00	1.00	7
v68	Sample size	0.82	0.97	17	0.75	1.00	8	0.89	0.74	9
v95, v96	results publication date (my)	0.87	0.97	15	0.86	0.95	7	0.88	0.97	8
	**Average**	**0.90**	**0.99**		**0.87**	**0.99**		**0.93**	**0.94**	

*Notes:* P_o = _percent agreement; ICC = intraclass correlation coefficient; N_subj_ = Number of compared subjects (i.e., surveys), LRD = Longitudinal research design, SP = Sampling procedure, IM = Interview mode. −9 (“No information“) and −7 (“No restriction”) excluded.

## Usage Notes

When working with the SDCCP dataset, we would like to highlight several points that data users should consider with respect to the structure, potential limitations, and linkage possibilities of our dataset.

First, individual cases (i.e., rows) are not necessarily independent of each other. Individual surveys or panel waves belong to larger survey contexts (e.g., survey programs). To enable data users to work with this clustered data structure, we included a set of ID variables (v2 - v5; see codebook). At the highest level, these variables enable a distinction between survey programs (v2), some of which consist of several sub-programs (v3). Again, each sub-program may consist of one or more surveys or waves (v4). Finally, for some surveys, several samples of a survey had to be recorded in separate rows (v5) if they differed in their survey design, and therefore, could not be coded together in a single row. Similarly, for some survey programs that included multiple surveys, only aggregated information that spanned multiple waves (e.g., total number of cases) was available. When we were unable to disentangle this information, we included such survey programs as a single row in the data set, and we added a technical variable (v6) indicating how many surveys or waves this entry technically covered. The decision to still include these cases was made to ensure the completeness of our records and to include as many eligible surveys as possible. As needed for their respective analyses, we recommend that data users utilize the information provided to account for the clustered structure of the SDCCP dataset.

Second, in our efforts to collect data about social science surveys conducted during the COVID-19 pandemic, we focused on surveys in Germany. This decision was made to offer in-depth insights in survey data collection practices during a time of crises within a similar context. Keeping the context (e.g., national legislation, funding opportunities) the same, enhanced the comparability between the surveys we included in our data. Focusing on Germany further enabled us to gather data to comprehensively study the data collection practices in an economically and politically important European country. Having said that, we are aware that the focus on Germany might impair the generalizability of our findings to surveys carried out in other countries. Therefore, we encourage similar research projects in other countries or even cross-nationally. Our research design, selection criteria, and coding scheme could serve as blueprints for such research. However, based on our own experience, we would caution researchers about the extent of the necessary personal resources for such an endeavor, and recommend they obtain appropriate funding.

Third, to select the surveys to be included in the SDCCP dataset, we developed and pursued a systematic approach. Thus, we relied on surveys that were searched for in the major German data archive for the social sciences, internationally renowned repositories in the same field, and the comprehensive RatSWD list, which we heavily relied on. This approach ensured that at least some publicly obtainable information was available to determine the eligibility of cases for inclusion in our dataset. To extend this list and include surveys that were not included in these lists and repositories, we conducted a web search that followed a snowballing method based on our earlier records. While we are confident that our method enabled us to include a major part of our target population, we would caution that not every survey is included in our dataset. We assume that especially smaller surveys that were conducted but did not publish their data for reuse or published them on private outlets are underrepresented in the data. Therefore, we recommend that data users interpret their findings accordingly and remember that smaller, less-well documented, and unpublished datasets likely are underrepresented in the SDCCP dataset.

Four, the SDCCP dataset can be linked with individual level survey data by using the technical variables on survey title and/or survey wave (in the case of panel surveys). Enriching existing datasets with SDCCP data can be useful when working with multiple survey datasets. In this case, survey design characteristics and our quality assessment can be considered in the analyses, for example, to control for the impacts of survey designs. For this purpose, design-based information that was coded as part of SDCCP can be utilized as a supplement.

**Fig. 3:**
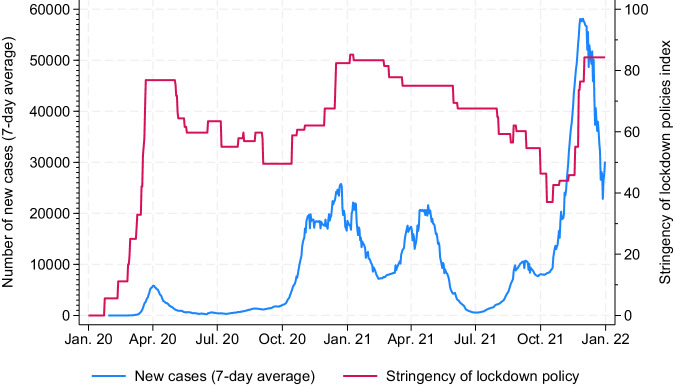
Contextual data on COVID-19 related indicators between 2020 and 2022 in Germany. Figure based on Oxford COVID-19 Government Response Tracker data^38^.

Fifth, furthermore, the SDCCP dataset can be supplemented with data on the events that occurred during the COVID-19 pandemic. This information can provide additional context when working with the SDCCP dataset. Figure 3 shows an example based on the data obtained from the Oxford COVID-19 Government Response Tracker^38^. For illustrative purposes, we plotted how the seven-day average of new COVID cases and an index of the stringency of lockdown policy developed between 2020 and 2022 in Germany. Information on the pandemic can be linked with the SDCCP dataset using the variables on the time of fieldwork.

## Data Availability

The code and data files that we used to assess interrater reliability (see section Technical Validation) are available at the GESIS Data Archive for the Social Sciences^39^.

## References

[1] Bavel JJV (2020). Using social and behavioural science to support Covid-19 pandemic response. Nat. Hum. Behav..

[2] Yamada Y (2021). COVIDiSTRESS Global survey dataset on psychological and behavioural consequences of the Covid-19 outbreak. Sci. Data.

[3] Blom AG (2020). High frequency and high quality survey data collection: The Mannheim Corona Study. Surv. Res. Methods.

[4] Kühne, S., Kroh, M., Liebig, S. & Zinn, S. The need for household panel surveys in times of crisis: The case of SOEP-CoV. *Surv. Res. Methods* 195–203, 10.18148/SRM/2020.V14I2.7748 (2020).

[5] Kapteyn A (2020). Tracking the effect of the Covid-19 pandemic on the lives of American households. Surv. Res. Methods.

[6] Kohler U (2020). Survey research methods during the Covid-19 crisis. Surv. Res. Methods.

[7] Cheng C (2022). Capturing the Covid-19 crisis through public health and social measures data science. Sci. Data.

[8] Gummer T (2020). The impact of Covid-19 on fieldwork efforts and planning in pairfam and FReDA-GGS. Surv. Res. Methods.

[9] Sastry N, McGonagle K, Fomby P (2020). Effects of the Covid-19 crisis on survey fieldwork: Experience and lessons from two major supplements to the U.S. Panel Study of Income Dynamics. Surv. Res. Methods.

[10] Burton J, Lynn P, Benzeval M (2020). How Understanding Society: The UK Household Longitudinal Study adapted to the Covid-19 pandemic. Surv. Res. Methods.

[11] RatSWD. Studien zur Corona-Pandemie. *KonsortSWD* https://www.konsortswd.de/themen/krisen/corona/ (2023).

[12] Dillman, D. A., Smyth, J. D. & Christian, L. M. *Internet, Phone, Mail, And Mixed-Mode Surveys: The Tailored Design Method*. (John Wiley & Sons, 2014).

[13] Groves, R. M. *et al*. *Survey Methodology*. (John Wiley & Sons, Hoboken, New Jersey, 2009).

[14] Post J, Class F, Kohler U (2020). Unit nonresponse biases in estimates of SARS-CoV-2 prevalence. Surv. Res. Methods.

[15] Schaurer I, Weiß B (2020). Investigating selection bias of online surveys on coronavirus-related behavioral outcomes. Surv. Res. Methods.

[16] Hipp L, Bünning M, Munnes S, Sauermann A (2020). Problems and pitfalls of retrospective survey questions in COVID-19 studies. Surv. Res. Methods.

[17] Page MJ (2021). The PRISMA 2020 statement: an updated guideline for reporting systematic reviews. BMJ.

[18] Lyberg, L. E. & Weisberg, H. F. Total survey error: a paradigm for survey methodology. in *The SAGE Handbook of Survey Methodology* 27–40 (2016).

[19] Groves RM, Lyberg L (2010). Total survey error: past, present, and future. Public Opin. Q..

[20] Biemer PP (2010). Total survey error: design, implementation, and evaluation. Public Opin. Q..

[21] Wilkinson MD (2016). The FAIR guiding principles for scientific data management and stewardship. Sci. Data.

[22] Wolf, C., Joye, D., Smith, T. W. & Fu, Y. *The SAGE Handbook Of Survey Methodology*. (SAGE, 2016).

[23] Marsden, P. V. & Wright, J. D. *Handbook Of Survey Research*. (Emerald Group Publ., Bingley, 2010).

[24] The American Association for Public Opinion Research. *Standard Definitions: Final Dispositions of Case Codes and Outcome Rates for Surveys. 10th Edition*. (2023).

[25] Jedinger A, Watteler O, Förster A (2018). Improving the quality of survey data documentation: a total survey error perspective. Data.

[26] Jabkowski P, Kołczyńska M (2020). Sampling and fieldwork practices in Europe: analysis of methodological documentation from 1,537 surveys in five cross-national projects, 1981–2017. Methodology.

[27] von Glasenapp, K., Skora, T., Gummer, T. & Naumann, E. SDCCP 1 - Survey Design and Data Quality During the Covid-19 Pandemic. 10.7802/2652. 10.1038/s41597-024-03475-x PMC11169371 38866799

[28] O’Connor C, Joffe H (2020). Intercoder reliability in qualitative research: debates and practical guidelines. Int. J. Qual. Methods.

[29] Feng GC (2014). Intercoder reliability indices: disuse, misuse, and abuse. Qual. Quant..

[30] Hallgren KA (2012). Computing inter-rater reliability for observational data: an overview and tutorial. Tutor. Quant. Methods Psychol..

[31] Klein D (2018). Implementing a general framework for assessing interrater agreement in Stata. Stata J..

[32] Brennan RL, Prediger DJ (1981). Coefficient Kappa: some uses, misuses, and alternatives. Educ. Psychol. Meas..

[33] Feng GC (2015). Mistakes and how to avoid mistakes in using intercoder reliability indices. Methodol. Eur. J. Res. Methods Behav. Soc. Sci..

[34] Byrt T, Bishop J, Carlin JB (1993). Bias, prevalence and Kappa. J. Clin. Epidemiol..

[35] Bennett EM, Alpert R, Goldstein AC (1954). Communications through limited-response questioning. Public Opin. Q..

[36] Landis JR, Koch GG (1977). The measurement of observer agreement for categorical data. Biometrics.

[37] Cicchetti D (1994). Guidelines, criteria, and rules of thumb for evaluating normed and standardized assessment instrument in psychology. Psychol. Assess..

[38] Hale T (2021). A global panel database of pandemic policies (Oxford COVID-19 Government Response Tracker). Nat. Hum. Behav..

[39] Skora, T. & von Glasenapp, K. Stata code for assessing interrater reliability in the coding of the SDCCP 1 dataset. 10.7802/2656.

